# Potential of metabolomics to reveal *Burkholderia cepacia* complex pathogenesis and antibiotic resistance

**DOI:** 10.3389/fmicb.2015.00668

**Published:** 2015-07-13

**Authors:** Nusrat S. Shommu, Hans J. Vogel, Douglas G. Storey

**Affiliations:** ^1^Biochemistry Research Group, Department of Biological Sciences, University of Calgary, Calgary, AB, Canada; ^2^Microbiology Research Group, Department of Biological Sciences, University of Calgary, Calgary, AB, Canada

**Keywords:** *Burkholderia cepacia* complex, virulence, antibiotic resistance, metabolomics, cystic fibrosis

## Abstract

The *Burkholderia cepacia* complex (Bcc) is a collection of closely related, genetically distinct, ecologically diverse species known to cause life-threatening infections in cystic fibrosis (CF) patients. By virtue of a flexible genomic structure and diverse metabolic activity, Bcc bacteria employ a wide array of virulence factors for pathogenesis in CF patients and have developed resistance to most of the commonly used antibiotics. However, the mechanism of pathogenesis and antibiotic resistance is still not fully understood. This mini review discusses the established and potential virulence determinants of Bcc and some of the contemporary strategies including transcriptomics and proteomics used to identify these traits. We also propose the application of metabolic profiling, a cost-effective modern-day approach to achieve new insights.

## *Burkholderia cepacia* Complex in Cystic Fibrosis

*Burkholderia cepacia* complex (Bcc) is a group of at least 17 Gram-negative β-*proteobacteria* that are phenotypically related but genetically discrete (Mahenthiralingam et al., 2005; Vanlaere et al., 2008, 2009). This complex of bacteria is resistant to many commonly used antibiotics and has a widespread distribution in nature existing in soil, water, plants, animals, and humans. During the last few decades *B. cepacia* complex bacteria have emerged as life threatening opportunistic pathogens especially in patients with cystic fibrosis (CF), the most common lethal inherited genetic disease among Caucasians (Isles et al., 1984). CF is caused by mutations in the gene encoding cystic fibrosis transmembrane regulator (CFTR), a transmembrane chloride ion channel (Kerem et al., 1989). The mutation in CFTR allows several opportunistic pathogens such as *Pseudomonas aeruginosa* and the *Burkholderia cepacia* complex, to colonize the lungs in CF patients leading to chronic infections. The Bcc bacteria are particularly troublesome because they can cause “Cepacia Syndrome,” a necrotizing pneumonia leading to a rapid deterioration of lung function, bacteremia and increased mortality (Isles et al., 1984). All 17 of the Bcc have been isolated from infected CF patients, however, the frequency and distribution of the Bcc in CF infections vary amongst the species (LiPuma, 1998; Mahenthiralingam et al., 2005).

Bacteria have to colonize the host mucosal or epithelial surfaces after entering the respiratory tract. Defective CFTR proteins in the CF lungs tends to dehydrate the mucus layer, which provides an ideal environment for biofilm formation (Matsui et al., 2006; Boucher, 2007). Several surface structures of the Bcc including adhesin, flagella and pili play crucial roles in motility and adherence to host cells (Urban et al., 2005). Secretion of extracellular proteins like lipases aid in invading epithelial cells (Mullen et al., 2007) whereas proteases aid in the proteolysis of the extracellular matrix, allowing the bacteria to colonize the lungs (McClean and Callaghan, 2009). Siderophore secretion allows bacteria to compete for iron with the host iron-binding proteins to support bacterial pathogenesis (Agnoli et al., 2006; Chu et al., 2010; Vogel, 2012). *Burkholderia* pathogens are known to use quorum sensing circuits for the regulation of various virulence factors including toxins, proteases, lipases and siderophores as well as for swarming motility and biofilm formation (Venturi et al., 2004; Eberl, 2006). Although there is no direct evidence of *in vivo* biofilm formation by Bcc bacteria in CF lung infections, isolates from a number of the different *Burkholderia* species have been shown to form biofilms *in vitro* (Conway et al., 2002). Interestingly, researchers have observed that Bcc produces mixed biofilms in cultures with *P. aeruginosa*, another prevalent CF pathogen (Riedel et al., 2001; Tomlin et al., 2001; Bragonzi et al., 2012). *P. aeruginosa* is frequently found with the Bcc in CF polymicrobial infections (Zemanick et al., 2011). Table 1 lists a number of classical screening approaches taken to identify a wide range of traits from the Bcc, involved in the physiology of the cells or virulence determinants.

**TABLE 1:** **Bcc physiologic and virulence traits identified by classical screening approaches**.

**Technique**	**Identified phenotypes**
Signature tagged mutagenesis	DNA replication and repair, global regulation, cellular metabolism and synthesis of cell surface proteins and polysaccharide (Hunt et al., 2004).
Plasposon mediated random mutagenesis	Cepacian, an exopolysaccharide required for biofilm formation, persistent infection and inhibition of host immune system (Cunha et al., 2003, 2004; Sousa et al., 2010).
Insertion mutagenesis	Hfq, an RNA chaperone regulator required for survival of the pathogen in CF patients (Sousa et al., 2010).
Insertion mutagenesis and computational techniques	Non-coding sRNAs interacting with Hfq chaperone (Ramos et al., 2011).
Screening infection model with mutant library	An acyl carrier protein (ACP) essential for colonization and metabolism of the pathogen in CF patients (Sousa et al., 2008).
	Pbr, a protein that plays a major role in bacterial stress tolerance and virulence mechanism (Ramos et al., 2010). In non-mammalian infection models CepR (the quorum sensing regulator), other regulatory and metabolic genes were isolated (Schwager et al., 2013).

One of the major concerns about Bcc pathogens is their intrinsic resistance to many commonly used antibiotics. The complex is resistant to a number of antibiotic classes including polymyxins, aminoglycosides, trimethoprim, chloramphenicol, quinolones, and β-lactams as well as to the host antimicrobial peptides (Nzula et al., 2002; Leitao et al., 2008; Sousa et al., 2011). The intrinsic multidrug resistance of Bcc bacteria is thought to occur due to the presence of various efflux pumps and enzymes that efficiently remove antibiotics from the cell, decreased contact of antibiotics with the bacterial cell surface due to their ability to form biofilms, and changes in the cell envelope that reduce the permeability of the membrane to the antibiotic (Buroni et al., 2009; Sousa et al., 2011; Rushton et al., 2013). It is also significant to note that biofilms produced by the Bcc are more resistant to antibiotics when compared to *P. aeruginosa* biofilms (Desai et al., 1998; Caraher et al., 2007; Dales et al., 2009). The multidrug resistance makes it extremely difficult to treat CF patients who are chronically infected with Bcc (George et al., 2009). Even though extensive knowledge has been accumulated on the virulence mechanisms and antibiotic resistance of *P. aeruginosa*, less is known about those mechanisms in Bcc. Profiling the metabolome of the Bcc, both extracellularly and intracellularly using a combination of high-resolution techniques like NMR, GC-MS, and LC-MS (Tang, 2011) can increase our understanding of these mechanisms.

## Potential of Metabolomics in the Study of BCC Pathogenesis

Metabolites incorporate the range of substances produced by cellular metabolism and signaling processes within an organism under any given physiological condition. A comprehensive understanding of the biological state can be achieved by studying the global metabolic response of any organism under natural or artificial environmental conditions (Tang, 2011). Improved application of analytical techniques such as nuclear magnetic resonance (NMR) and mass spectrometry (MS) combined with gas chromatography (GC) or liquid chromatography (LC) has enabled the synchronized detection and comparison of a wide range of metabolites in biological systems (Fiehn, 2002). Metabolic profiling has been applied to microbes for a wide range of studies including genome annotation and pathway mapping, host-microbe interactions, infectious disease research, drug metabolism, heavy metal resistance as well as many environmental and ecological analyses (Chen et al., 2007; Halouska et al., 2007; May et al., 2008; Parisi et al., 2009; Slupsky et al., 2009; Kwon et al., 2010; Booth et al., 2011; Donovan et al., 2012; Ye et al., 2012; Mickiewicz et al., 2013, 2014; Xie et al., 2013). For instance Fang et al. (2011) have used gene annotation and bioinformatics to construct a genome scale metabolic network for *B. cenocepacia* J2315. Bartell et al. (2014) using a similar approach have compared the metabolic networks of *B. cenocepacia* to *B. multivorans*, the two *Burkholderia* species most frequently isolated from CF patients. However, what is now needed is a systematic approach to examine the metabolome of both *B. cenocepacia* and *B. multivorans* under conditions that mimic the CF lung environment, such as growth in a defined synthetic media as was used for *P. aeruginosa* (Kozlowska et al., 2013).

Metabolites from biofluids (blood, plasma, urine etc.) of patients or model organisms are analyzed to detect significant metabolites that could be used as an indicator of the infection. For instance, by applying a ^1^H NMR metabolomics approach researchers have been able to identify biomarkers that could be useful for early detection of sepsis, a life threating infectious disease (Mickiewicz et al., 2013, 2014). Moreover, metabolic profiling has successfully distinguished mice with Gram-positive bacterial infection from those with Gram-negative infection (Hoerr et al., 2012). Clearly, metabolomics is gaining notable popularity in the studies of infectious pathogens as well as the resulting disease conditions.

We envision that the human airway epithelial cell line A549 could be an option for *in vitro* metabolomics experiments. Although A549 is not a CF cell line, it has been used to measure invasion of *B. cepacia* in an antibiotic protection assay and the results showed correspondence with another invasion study in a mouse agar bead infection model (Burns et al., 1996; Cieri et al., 2002). The mouse model will be preferable for *in vivo* experiments as *Cftr*^–/–^ knockout mice (*Cftr*^m1HGU^ or *Cftr*^m1UNC^) mice are a widely used infection model for CF studies however, considering the rapid clearance of the Bcc bacteria from mice lungs, this model can only be used to study short-term but not chronic infections (Davidson et al., 1995; Sajjan et al., 2001). One of the most useful models for chronic CF infection is the agar bead model, in which Bcc bacteria persist in the lungs for as long as 21 days. In this infection model Bcc bacteria fixed into agar beads are injected into mice or rats (Cieri et al., 2002; Subsin et al., 2007). We propose initially using A549 cells and looking at the metabolome of invasive *Burkholderia cepacia* in tissue culture as compared to cells grown in a rich media. Schoen et al. (2014) used a similar approach on invasive *Neisseria meningitidis* and showed how meningococcal metabolism is linked to pathogenesis (Schoen et al., 2014). The research could then progress to animal models and eventually to human infections. For human infections we propose using Bronchoalveolar lavage samples from CF patients with *Burkholderia* infections to determine the metabolomics of the bacterial lung population. A number of technical hurdles would have to be overcome but such an approach has been applied on lavage samples from HIV infected patients (Cribbs et al., 2014).

In the context of the Bcc, only a few studies using metabolomic approaches have been carried out to date. In one of these studies the researchers employed metabolic profiling to compare the mechanisms of osmotic tolerance among five closely related isolates of *B. cenocepacia*, a clinically and environmentally significant member of *B. cepacia* complex (Behrends et al., 2011). Five strains of *B. cenocepacia* namely C1394, CEP0511, J2315, J415, and K56-2 are grown in rich medium with a sodium chloride (NaCl) concentration similar to that of seawater, resulting in severe osmotic stress leading to impaired growth for all the strains. Although each strain grows in identical salt concentration, the extent of osmotic tolerance varies; J415 and K56-2 exhibit more resistance than C1394, CEP0511, and J2315. In order to get an insight into the differential effect of osmotic stress on the strains the intracellular metabolites have been profiled using NMR spectroscopy. Five potential osmo-responsive metabolites: alanine, phenylalanine, glutamate, trehalose, and glycine-betaine have been detected. Relative quantification of these five metabolites indicated that the semi osmo-tolerant strain K56-2 has increased levels of all the five osmoprotectants, the three less tolerant strains C1394, CEP0511, and J2315 do not exhibit a significant increase in trehalose and glycine-betaine levels, and the other semi-tolerant strain J415 show only slightly induced trehalose level under osmotic stress. This study demonstrates that metabolomics could help identify the different strategies of osmotic tolerance amongst isolates of the same species and even the same lineage (Behrends et al., 2011). We are in agreement with these authors that future metabolic experiments should look at physiological concentrations of NaCl, which would have more relevance to CF lung infections. Again this approach could also be applied to *B. multivorans* and perhaps to other members of the Bcc that infect CF patients.

Although evidence of metabolomics studies on pathogenesis of Bcc are somewhat scarce, some successful transcriptomics and proteomics based studies have been reported. The group of Yoder-Himes has compared the gene expression of a CF isolate of *B. cenocepacia* and its environmental counterpart. In spite of a high genomic sequence resemblance between the isolates, the study identified 458 strain-specific genes, 126 clinical-isolate-specific, and at least four species-specific genes that are stimulated in the CF pathogen suggesting the potential of these genes to serve as novel drug targets (Yoder-Himes et al., 2009, 2010). Comparative transcriptomics has been applied to isolates of the same *B. cenocepacia* strain recovered from CF patients at different stages of chronic infection and almost 1000 genes involved in translation, iron acquisition, efflux of drugs and adhesion to respiratory epithelial surface are found to be induced in the course of long-term infection (Mira et al., 2011). Finally, the proteomic profile of a *B. cenocepacia* isolate that exhibits prolonged persistence in CF patients has been compared with that of another isolate from the same species showing rapid clearance from the host; the study suggests that higher expression of flagellin protein in the persistent strain provides enhanced mobility to support survival in CF patients (Chung and Speert, 2007). As metabolite levels reflect the ultimate response of any biological system, integration of metabolomics to these “-omics” approaches would enrich the current understanding of Bcc pathogenesis. For instance, comparative metabolic profiling of the pathogenic strain of *B. cenocepacia* and its environmental counterpart in conditions that would mimic the CF lung environment and soil would confirm and extend the findings of Yoder-Himes (Yoder-Himes et al., 2009, 2010). Similarly, application of metabolic profiling to Bcc strains having different persistent capacities or collected at different stages of infection as done by Chung and Speert (2007) and Mira et al. (2011) respectively, would add insight to which type of metabolism each isolate was using during chronic infection. Such a metabolic analysis would also reveal any adaptation of the metabolism of the bacteria during an infection.

Metabolite profiling has recently been applied to study the virulence of *P. aeruginosa* in CF lung infections. As the Bcc and *P. aeruginosa* share similar biology, the studies on *P. aeruginosa* could essentially guide the study of metabolic profile underlying the virulence and antibiotic resistance of *B. cepacia* complex. Using clinical isolates and mutant libraries researchers have been able to characterize the metabolites involved in metabolic adaptation, biofilm formation and antibiotic resistance of *P. aeruginosa* in CF patients (Behrends et al., 2013a,b; Borgos et al., 2014). In one study the research group of Behrends assessed the metabolic alteration of *P. aeruginosa* during chronic infection using NMR spectroscopy (Behrends et al., 2013b). They isolated 179 strains from 18 chronic CF patients with infection periods ranging from 4 to 24 years so that the strains epitomize clonal lineages from these individual patients. They compared clonal sequential isolates grown in rich LB media for 24 h, profiled the extracellular metabolites and detected eight significant compounds involved in metabolic adaptation. Six amino acids—phenylalanine, tryptophan, tyrosine, valine, lysine and serine and the metabolic intermediate acetate have demonstrated negative correlation with length of the infection. On the other hand, the secretion of osmoprotectant trehalose escalated with the duration of infection (Behrends et al., 2013b). The same group later studied the extracellular metabolites of eighty-six single-gene mutant strains of *P. aeruginosa* grown in nutritional conditions that mimicked CF sputum (Behrends et al., 2013a). One remarkable finding of this NMR study is that discharge of excessive amounts of gluconate by a number of mutant strains correlates with reduced susceptibility to antibiotics tobramycin, ciprofloxacin, aztreonam and imipenem, indicating a direct relevance of gluconate accumulation with antibiotic resistance of many pathogenic *P. aeruginosa* strains (Behrends et al., 2013a). Recently another research group has applied high resolution MS fingerprinting to study the metabolic profile underlying *P. aeruginosa* biofilm formation during opportunistic infection (Borgos et al., 2014). Extracellular biofilm compounds analyzed from *in vitro* cultures of four different *P. aeruginosa* strains at different time points have revealed strain and time dependent changes in the metabolic profiles. The interesting outcomes from the metabolic studies of *P. aeruginosa*, and the close link between these species during opportunistic CF infection, suggest that carrying out similar experiments on the Bcc could lead to important findings on virulence and antibiotic resistance of these pathogens in CF infection. To support these studies Bcc mutants in metabolism genes have been reported by many labs some of which are outlined in Table 1. However, transposon or plasposon mutagenesis could also be used to generate additional mutants (Dennis and Zylstra, 1998; Winson et al., 1998). In this case the mutants could be compared to the parental strain in terms of changes in metabolites. Finally, as many intermediate and end products of central metabolism remain inside the cell, analysis of intracellular along with the extracellular metabolites would provide a more comprehensive insight into the metabolic changes in the Bcc.

## Conclusion

During the past few decades Bcc has emerged as a group of deadly pathogens particularly to CF patients. Tolerance to most of the commonly used antibiotics remains a challenge to combat this opportunistic pathogen. Improved techniques for gene identification, access to a plethora of genomic information and the development of multiple useful *in vivo* and *in vitro* infection models have formed a good foundation toward understanding the virulence of these bacteria; however, a clearer understanding is needed to acquire comprehensive insights into the pathogenesis and antibiotic resistance of the Bcc species. Metabolomics, along with other systems biology techniques and conventional approaches should be adopted to study the global view on the virulence mechanisms of Bcc. As metabolites reflect the ultimate interactions between the organism and its environment, the metabolic profile of the Bcc could aid in revealing an overall state of the bacteria in the context of virulence in CF lungs, as well as its tolerance to different antibiotic treatments. Ultimately these approaches could reveal new information, which might be useful for the design of novel drugs against chronic CF infections.

### Conflict of Interest Statement

The authors declare that the research was conducted in the absence of any commercial or financial relationships that could be construed as a potential conflict of interest.
